# Prophylactic onlay mesh at emergency laparotomy: promising early outcomes with long‐acting synthetic resorbable mesh

**DOI:** 10.1111/ans.17925

**Published:** 2022-08-01

**Authors:** Daniah Alsaadi, Ian Stephens, Lydia O. Simmons, Magda Bucholc, Michael Sugrue

**Affiliations:** ^1^ Department of Surgery Letterkenny University Hospital Letterkenny Ireland; ^2^ Intelligent Systems Research Centre, School of Computing, Engineering and Intelligent Systems Ulster University Derry UK; ^3^ Donegal Clinical Research Academy Letterkenny University Hospital Letterkenny Ireland

**Keywords:** burst abdomen, emergency laparotomy, incisional hernia, patient outcomes, prophylactic mesh placement, seroma, surgical site infection, TIGR

## Abstract

**Background:**

Careful surgical strategy is paramount in balancing the prevention of fascial dehiscence, incisional hernia (IH) and fear of additional mesh‐related wound complications post‐laparotomy. This study aims to review early outcomes of patients undergoing an emergency laparotomy with prophylactic TIGR® mesh, used to reduce early fascial dehiscence and potential subsequent IH.

**Method:**

A retrospective, ethically approved review of 24 consecutive patients undergoing prophylactic TIGR® mesh placement during emergency laparotomies by a single surgeon between January 2017 and June 2021 at a University Hospital. A standardized approach included onlay positioning of the mesh, small‐bite fascial closure, and a wound bundle. We recorded patient demographics, operative indications, findings, degree of peritonitis, postoperative complications, and mortality.

**Result:**

The study included 24 patients; 16/24 (66.6%) were female and median age was 72.5 (range 31–86); 14/24 patients were ASA grade III or greater; 4/24 patients (16.6%) developed six complications and 3/6 occurred in a single patient. Complications included subphrenic abscess, seroma, intrabdominal hematoma, enterocutaneous fistula leading to deep wound infection and small bowel perforation. Five (20.8%) patients died in hospital; central venous catheter sepsis (*n* = 1), fungal septicaemia (*n* = 1) and multiorgan failure (*n* = 3). Surgical site infection and seroma rates were low, occurring in 2/24 patients (4% each).

**Conclusion:**

This study has identified that prophylactic onlay mesh in patients undergoing an emergency laparotomy is not associated with significant wound infection or seroma when used with an active wound bundle. The wider use of TIGR® to prevent fascial dehiscence and potential long‐term IH prevention should be considered.

## Graphical abstract

Careful surgical strategy is paramount in balancing the prevention of fascial dehiscence, incisional hernia and fear of additional mesh‐related wound complications post‐laparotomy. This study aims to review early outcomes of patients undergoing an emergency laparotomy with prophylactic synthetic TIGR® mesh, used to reduce early fascial dehiscence and potential subsequent incisional hernia.

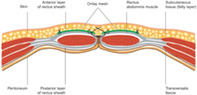

## Introduction

Fascial dehiscence, early as a burst abdomen and later as an incisional hernia (IH), is recognized as significant potential sequelae of both elective and emergency abdominal surgery occurring in 2–30% of cases.^1^ Over 4 million laparotomies are performed annually in the United States alone with an average IH rate of 20%, resulting in approximately 800 000 new IHs each year.^2^, ^3^ Abdominal fascial dehiscence has been reported to occur in 2%–10% of emergency laparotomies, with an associated postoperative mortality rate of 20%–45%.^4^

For over a decade, various evidence‐based interventions have been shown to reduce IHs, including wound bundles, optimal surgical fascial closure techniques and prophylactic mesh placement (PMP).^5^ Wound bundles, a relatively recent innovation in surgery, are a series of evidence‐based interventions known to improve patient outcomes when combined and have a positive impact on both elective abdominal surgeries and emergency laparotomies.^6^, ^7^ Simple techniques, including the documentation of suture‐to‐wound length ratios during fascial closure and the use of small bite sutures, have been shown to reduce IH rates by 30%.^8^ Recent randomized controlled trials have shown PMP to be beneficial during fascial closure, reducing IH rates by at least half.^1^, ^2^, ^9^, ^10^ There are numerous potential positions for mesh placement: onlay, inlay, sublay, underlay and intraperitoneal, each with their own benefits and complication profiles.^11^ Onlay mesh placement was popularized by Chevrel in 1997 and is technically easier to perform.^12^

While studies of onlay macroporous Prolene®^1^ mesh have been promising in preventing IH, surgeons have reported adverse effects following mesh insertion, including surgical site infection (SSIs) and mesh extrusion. This has led to a reluctance to change surgical practices.^13^, ^14^ In theory, long‐acting resorbable (LAR) synthetic meshes offer potential advantages that may reduce these concerns.^15^ The use of TIGR®,^2^ a multifilament bio‐absorbable mesh, has not been widely reported outside breast surgery despite being available for the last 20 years.^16^, ^17^ This study reviews the early outcomes of patients undergoing prophylactic TIGR® mesh placement in abdominal closure during emergency abdominal surgery.

## Methods

A retrospective, ethically approved review of all patients who had prophylactic TIGR® mesh placement during emergency abdominal surgery was undertaken at Letterkenny University Hospital between January 2017 and June 2021. The patients' demographics, operative indications, findings, and degree of peritonitis were collected. The Mannheim Peritonitis Index (MPI), interpreted predicted mortality rate, and World Society of Emergency Surgery (WSES) scoring system were calculated.^18^, ^19^ The Portsmouth physiological and operative severity score for mortality and morbidity (P‐POSSUM) and the National Emergency Laparotomy Audit (NELA) prediction model were calculated using the Risk Predication in Surgery P‐POSSUM calculator and NELA risk calculator. Mortality was defined as in‐patient death or death within 30 days of the index operation. The Center for Disease Control and Prevention (CDC) surgical wound grading system (I: clean; II: clean/contaminated; III: contaminated; IV: dirty) was used to classify surgical wounds during the operation.^20^ Clavien‐Dindo classification was used to record postoperative complications.^21^ There were three patients who had damage control surgery during the study period. These were not included in this study as they did not have a mesh. Four other patients did not have mesh inserted due to its lack of availability.

Surgery was performed by a single consultant surgeon (MS), and fascial closure was performed using a small bite technique with a 5 mm separation of continuous 2/0 polypropylene (Prolene®)^3^ and a 10 mm‐wide bite in the fascia. This was in line with the small bite small suture STITCH study approach.^8^ The preference of the team was to use a non‐absorbable, although an absorbable suture could be used. Before fascial closure, 4 cm of subcutaneous fat was dissected from the fascia on either side of the midline. It was the surgeon's routine practice to place the mesh. TIGR® mesh was placed in an onlay position, as shown in Figure S1, using two 4 cm‐wide strips on either side of the fascia prior to fascial closure. The mesh was fixed to the fascia using a 2/0 continuous Prolene® suture. The fascia was then closed, and the mesh was incorporated into the primary closure. The subcutaneous tissue was closed in layers interrupted with a 3/0 Polydioxanone Suture (PDS) stitch with the bite incorporating the mesh to bring the subcutaneous tissue down to the mesh plane to ensure it was closed. Skin closure was performed using subcuticular 3/0 Stratafix®,^4^ except in two patients with fasciitis where the skin was left open and an ActiVAC™^5^ negative‐pressure wound therapy (NPWT) dressing system was applied. In the two patients who had the skin left open this was a delayed closure of the dermis however the subcutaneous tissue was closed in a similar fashion. The time taken for fascial exposure and onlay placement was not recorded but, in our experience, the process takes 20–25 min. All patients were followed up and reviewed as outpatients, and the wounds were assessed for SSIs and clinical evidence of IHs.

Designated wound bundles were used in all patients with preoperative prophylactic antibiotics, including optimization of blood glucose <7.8 mmol, prevention of hypothermia and hair removal in the operating room. Intraoperatively, skin preparation with 2% chlorhexidine gluconate in 70% isopropyl alcohol, double gloving, abdominal wound protector, a change of instrument tray for abdominal closure, quilting subcutaneous layers, glucose control, prevention of hypothermia and intraperitoneal irrigation with a solution containing 240 mg of gentamycin and 600 mg of clindamycin were practiced.^22^ Postoperatively, patient wound care advice, wound inspection and the prophylactic use of PICO®^6^ NPWT dressings on high‐risk patients were performed. The NPWT dressings were left intact until day five unless there was contamination of the dressing. Patients and their wounds were reviewed at 2 weeks post‐discharge.

## Results

This study identified 24 consecutive patients who underwent an emergency laparotomy with prophylactic onlay mesh placement. Their median age was 72.5 years (range 31–86) and 16/24 (66.6%) were females. Demographic variables, including American Society of Anaesthesiologists' (ASA) grades, risk factors and wound classification are shown in Table S1.

In total, 22/24 (87.5%) patients had a bifid incorporating prophylactic mesh placement with strips fixed to the fascia on each side before primary fascial closure. Two patients (8%) had fascial closure with a single strip onlay mesh placed on top of the primary fascia closure. Six (25%) patients underwent multiple operations (range: 2–6) during their admission. The prophylactic mesh was placed during the last surgery in four patients. Two patients had mesh placed during the initial surgery and required subsequent unplanned re‐operation for intrabdominal complications unrelated to the mesh. The mesh was removed in one of these cases (4%). The skin was closed primarily in 23/24 (96%) patients. The preoperative risk assessment and admission physiology for patients who had mesh inserted are shown in Table 1. The operative indications and name of surgeries are shown in Table S2.

**Table 1:** Preoperative risk assessment

Patient (*n* = 24)	Age	Gender	LOS (days)	Systolic blood pressure	Pulse rate	pH	C‐Reactive protein (CRP)	Mannheim Peritonitis Index score	Mortality prediction rate %	WSES score	P‐POSSUM mortality %	Possum morbidity %	NELA mortality%
	46	F	9	137	91	7.4	4	9	20	0	1.7	37.8	0.4
	55	F	6	85	68	7.39	22.4	18	13	7	7.1	74.3	9.3
	74	F	9	138	95	7.38	320	10	20	5	14.3	88.2	2.9
	83	F	10	157	94	7.28	61	10	20	5	9	73.9	3.3
	57	F	6	133	53	7.4	4	10	20	0	0.8	20.4	1.2
	31	M	8	118	98	7.29	71	10	20	3	2.3	48.5	0.5
	37	M	8	148	85	7.41	4	0	0	0	2.1	40.1	0.2
	78	M	10	167	95	7.33	22	5	0	2	4.7	60.6	6.6
	44	M	220	135	63	7.43	111	0	0	5	2	43	0.2
	56	F	41	131	81	7.41	8	24	26	0	2	42.3	0.7
	86	F	11	112	87	7.43	8	20	13	5	48.9	97	16.8
	75	M	19	130	50	7.43	510	5	0	7	10.6	84.4	5.1
	84	F	13	148	108	7.39	90	10	20	5	4.1	56	6.2
	67	F	8	116	80	7.37	16.9	10	20	0	1.1	26.1	1.4
	32	M	10	149	110	7.34	26	16	13	3	13.6	89.4	2.5
	70	F	6	135	81	7.4	10.5	10	20	2	1.8	36.4	1.3
	71	F	12	186	90	7.4	8.12	10	20	5	6.3	69.2	5.2
	83	M	36	154	76	7.49	23	5	0	5	10	78.9	9.4
	77	F	9	166	108	7.45	411	10	20	2	8.6	76.1	3
	84	F	11	140	98	7.3	343	27	26	9	26	92.4	46.9
	69	F	11	130	78	7.3	147	14	13	10	14.5	87.9	28.7
	81	F	33	134	73	7.39	431	20	13	9	8.3	77.2	5.3
	78	M	23	140	76	7.48	5.9	27	26	6	7.1	74.3	10.3
	79	F	14	134	65	7.44	411	20	13	10	10	78.9	11.7
Median (Range)	72.5 (31–86)		10.5 (6–220)	136 (85–186)	83 (50–110)	7.4 (7.28–7.49)	66 (4–510)	10 (0–27)	20 (0–26)	5 (0–10)	7.1 (0.8–48.9)	74.1 (20.4–97)	4.2 (0.2–46.9)

*Note*: Patients 20–24 died.

The median operation duration was 195 min (range: 85–305 min), and the median length of hospital stay was 10.5 days (range: 6–220 days). One patient remained as an inpatient for 220 days due to an enterocutaneous fistula related inflammatory bowel disease (IBD). Of the 24 patients, 12 (50%) were treated in the intensive care unit and the remainder in the high dependency unit.

Four of the 24 patients (16.6%) developed six complications and 3/6 complications occurred in a single patient, comprising an enterocutaneous fistula and an associated deep wound infection along with small bowel perforation. This complex colitic patient with IBD, following an emergency subtotal colectomy, developed a small bowel perforation requiring re‐laparotomy on day five. During the second operation for peritonitis secondary to the small bowel perforation, a resection with anastomosis was performed, and the original onlay TIGR® mesh was removed at the start of the procedure. Five days later, an anastomotic small bowel leak was detected, leading to an open abdomen and a prolonged anastomotic fixed deep enterocutaneous fistula with an associated deep wound infection. To achieve delayed primary fascial abdominal closure and prevent dehiscence, a macroporous onlay Prolene® mesh was used to prevent abdominal dehiscence. Four weeks later, the mesh was removed to facilitate granulation of the open wound. A subphrenic abscess, seroma and intrabdominal hematoma occurred separately in three individuals. The seroma was managed with percutaneous aspiration. The Clavien–Dindo classification scores were noted as follows: I (*n* = 1), III (*n* = 3) and V (*n* = 5).

Five of the 24 (20.8%) patients died in the hospital: patients 20–24 in Tables 1 and S2. Causes of death included central venous catheter sepsis (*n* = 1), fungal septicaemia (*n* = 1) and multiorgan failure secondary to sepsis (*n* = 3). The cause of death was established by post‐mortem examination. Central venous catheter sepsis was determined from blood cultures and the tip of the catheter. Care was withdrawn from patients 20, 21 and 23 following multidisciplinary discussions with their families. The median time to follow up in the outpatient clinic after discharge from the hospital was 8 weeks (range: 2–31.5 weeks). Subsequently, patients were referred back to their general practitioners.

## Discussion

This single‐surgeon series of high‐risk patients has provided insight into the use of LAR mesh, TIGR®, in emergency surgery patients with an onlay mesh placement technique. This study, while retrospective, is consecutive and reflects real‐world emergency surgery practices; while the number of patients studied is relatively small, they represent typical complex emergency general surgery patients. The patients, with two exceptions, had serious abdominal infections or ischaemia in the presence of major comorbidities. Fourteen out of 24 (58%) patients were ASA III with expected significant morbidity and mortality that varied depending on the scoring system used. Moreover, 10/24 (41.6%) patients had contaminated wounds (*n* = 3) and infected wounds (*n* = 7), and one‐third of the patients were obese. Based on the cohort in this study, the risk of abdominal wall complications should approach 30%, wound dehiscence 10% and subsequent IH rates of 30% at 5 years.^1^, ^23^ The extra time and expense required for the use of PMP in these patients must be justified and one could argue that, in the five patients who died, unless a burst abdomen was prevented, the prophylactic mesh constituted overtreatment.

Eighteen (75%) patients in this series had gastrointestinal‐related large and small bowel perforations. Five (20.8%) patients underwent resections with primary anastomosis, and the use of open abdomen only occurred in four patients. Given the potential for the P‐POSSUM to overestimate mortality, we used the NELA score,^24^ which, depending on the patient cohort, ranges from 0.2% to 46.9%. The actual mortality for the current series was 20.8% greater than the NELA and P‐POSSUM predicted mortality median rates of 4.2% and 7.1%, respectively. Due to the heterogenicity of patients, mortality comparison with other emergency laparotomy series who report a mortality rate of 9.5%–18.4% is difficult.^23^ The mesh did not contribute to the mortality rate in our study. The seroma rate of 4% was less than reported in some studies and may reflect the combined use of dead space subcutaneous closure and incisional NPWTs.^17^ The use of quilting and a comprehensive wound bundle with careful tissue handling, we believe, accounts for the low rate in our series. It is now recommended that in all colorectal surgery that a wound bundle with appropriate antibiotic be used to reduce risk of infection.^25^

Fascial closure techniques have been the focus of multiple studies with paradigm shifts from mass closure to the small bite technique. In the STITCH trial, conducted predominantly on elective patients, 5–5 separation and width bites were recommended.^8^ Small bite closure has not yet gained traction among surgeons in either elective or emergency laparotomy closures. Reasons for this include closure‐related complications, unfamiliarity with the method, long execution time, patient criteria, and width and strength of the sutures.^14^

In our study, patients underwent a modified small STITCH approach with a 5 mm separation with a 10 mm width based on evidence that a wider bite may be more beneficial.^26^ The authors would now recommend the use of 3/0 PDS to fix the mesh rather than 2/0 Prolene® as this would be absorbed. Chevrel's classic onlay mesh technique places and fixes the mesh after fascial closures.^12^ Our paper proposes a new technique where the mesh is placed in two lateral strips, acting as a buttress to the fascia closure and offering theoretical pledget‐type support to prevent dehiscence and hopefully reduce IH.

While original studies of mesh insertion were conducted on both clean and clean‐contaminated laparotomy wounds, there is increasing evidence that they are not associated with increased SSI.^27^, ^28^ The PRIMA trial provided level‐one evidence that PMP reduces rates of IHs with a mesh removal rate of 10.2%. In 10 out of 373 (2.7%) patients, the mesh was removed in consequence to infection.^13^ Similarly, a recent randomized clinical trial confirmed the efficacy and safety of PMP in high‐risk patients undergoing emergency laparotomies. Seven out of 52 (13.5%) patients who had fascial closures without mesh reinforcement developed fascial dehiscence compared to zero in the group of patients that received a mesh.^29^

LAR (i.e. biodegradable, bioabsorbable and biosynthetic) meshes are the most recent development in this area. These products are composed of synthetic polymers that serve as a scaffold for host–tissue ingrowth. Native collagen replaces the mesh as it slowly degrades, theoretically averting the potential for chronic infection. Currently, there are three LARs on the market: TIGR® matrix, Gore® Bio‐A®^7^ and Phasix Mesh™^.^ ^8^ TIGR® matrix surgical mesh is composed of two different synthetic resorbable fibres. The first fibre, which constitutes 40% of the matrix, is a copolymer of polyglycolide, polylactide and polytrimethylene carbonate. The second fibre, which constitutes 60% of the matrix, is a copolymer of polylactide and polytrimethylene carbonate. Both fibres are degraded by bulk hydrolysis, with the loss of fibres resulting in a decreasing tensile strength.^15^ Ultimately, the argument for LARs is that they provide the benefits of a biologic material at a lower cost. A value‐based clinical quality improvement project for patients undergoing abdominal wall reconstruction (AWR) showed that the TIGR® matrix was better value compared with the published results of biologic mesh, with similar outcomes observed and decreased mesh costs. Consistent durability of repair was confirmed by long‐term follow up.^17^

Finally, our study was somewhat limited as the number of patients was relatively small, a single surgeon was used and the study was retrospective and non‐comparative. Future randomized clinical studies compromised of larger sample sizes and long‐term follow up are warranted.

## Conclusion

This study has identified that prophylactic onlay mesh in patients undergoing emergency laparotomy is not associated with significant wound infection or seroma when used with an active wound bundle. No fascial dehiscence was seen. The wider use of TIGR® to prevent early and late fascial dehiscence could be considered.

## Author contributions

**Daniah Alsaadi:** Conceptualization; data curation; methodology; writing – original draft; writing – review and editing. **Ian Stephens:** Supervision; writing – review and editing. **Lydia O. Simmons:** Supervision; writing – review and editing. **Magda Bucholc:** Writing – review and editing. **Michael Sugrue:** Conceptualization; writing – review and editing.

## Funding information

This project was partly supported by the European Union's INTERREG VA Programme, managed by the Special EU Programmes Body (SEUPB) and Donegal Clinical and Research Academy.

## Conflict of interest

Michael Sugrue has received speaker fees from Acelity, Smith and Nephew and Novus Scientific. The other authors declare that they have no competing interests. No funding was received from Novus Scientific for this study, and the mesh used was procured following normal hospital procurement practices.

## Ethical approval

Ethical approval was obtained from Letterkenny University Hospital's Ethics Committee.

## Supporting information


**Figure S1.** Mesh placement

**Table S1.** Patient demographics and wound classification
**Table S2.** Indications and operations where mesh was inserted

## Data Availability

The datasets supporting the conclusions of this article are included within the article and the supplementary section.

## Endnotes

^1^
Ethicon, Somerville, New Jersey, USA.

^2^
Novus Scientific, Uppsala, Sweden.

^3^
Ethicon, Somerville, New Jersey, USA.

^4^
G.F. Mersons Limited, Ethicon Suture Laboratories.

^5^
3 M KCI, San Antonio, TX, USA.

^6^
Smith and Nephew Healthcare, Hull, United Kingdom.

^7^
WL Gore and Associates, Inc., Flagstaff, Ariz.

^8^
CR Bard, Inc./Davol Inc., Warwick, R.I.
