# Supplementary figures and images for: Prophylactic onlay mesh at emergency laparotomy: promising early outcomes with long‐acting synthetic resorbable mesh
Source: ANZ J Surg. 2022 Aug 1;92(9):2218–23. doi: 10.1111/ans.17925 (PMC9540974; doi:10.1111/ans.17925)

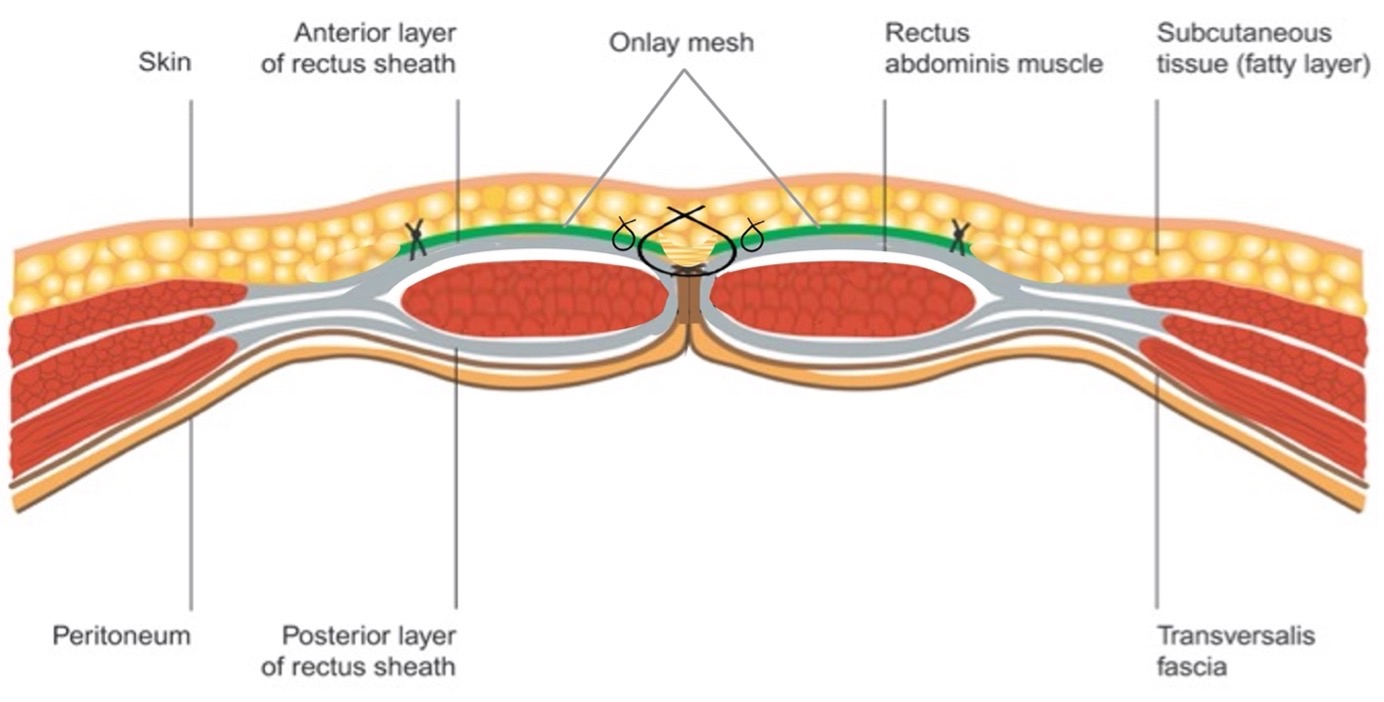

Supplement: Supplementary file 1 — Figure S1. Mesh placement [file ANS-92-2218-s002.jpg]
