# Supplementary material for: Prophylactic onlay mesh at emergency laparotomy: promising early outcomes with long‐acting synthetic resorbable mesh
Source: ANZ J Surg. 2022 Aug 1;92(9):2218–23. doi: 10.1111/ans.17925 (PMC9540974; doi:10.1111/ans.17925)
Supplement: Supplementary file 2 — Table S1. Patient demographics and wound classification Table S2. Indications and operations where mesh was inserted [file ANS-92-2218-s001.docx]

**Supporting Information**

**Table S1.** Patient demographics and wound classification

| **Variables** | **Patients (n=24)** |
| --- | --- |
| ASA (*n*) |  |
| I | 1 |
| II | 9 |
| III | 10 |
| IV | 3 |
| V | 1 |
| Risk factors |  |
| BMI |  |
| >35 | 3 |
| >30 | 5 |
| >25 | 7 |
| <18.5 | 1 |
| Smoking | 4 |
| Respiratory disease | 7 |
| Diabetes | 4 |
| Comorbidities | 13 |
| Previous abdominal surgery | 8 |
| Wound classification |  |
| II | 14 |
| III | 3 |
| IV | 7 |

| **Table S2.** Indications and operations where mesh was inserted | | |
| --- | --- | --- |
| **Patient**  **(n=24)** | **Operation** | **Indication** |
|  | Laparoscopic cholecystectomy converted to open | Gangrenous cholecystitis with 7 previous laparotomies |
|  | Repair of strangulated umbilical hernia + ovarian cystectomy (perforated) | Strangulated umbilical hernia |
|  | Subtotal colectomy and end ileostomy | Fulminant colitis |
|  | Jejunal resection for small bowel obstruction ischaemia | Ischaemic bowel |
|  | Laparotomy and repair of internal small bowel hernia | Small bowel obstruction from internal herniation of jejunal loop |
|  | Left hemicolectomy | Megacolon with abdominal compartment syndrome |
|  | Laparotomy and resection of small bowel and adhesions | Crohn's small bowel stricture |
|  | Obstructing sigmoid cancer resection with anastomosis | Obstructing sigmoid cancer |
|  | Subtotal colectomy and ileostomy | Fulminant ulcerative colitis |
|  | Primary repair of perforated gastric ulcer | Perforated gastric ulcer |
|  | Open appendicectomy | Appendicular mass and grade V appendicitis |
|  | Relook laparotomy and abdominal wall closure | Hinchey III Diverticulitis |
|  | Laparotomy and small bowel resection | Small bowel obstruction and mesenteric ischemia |
|  | Extended sigmoid colectomy with primary anastomosis | Obstructing sigmoid cancer |
|  | Pancreatic Roux-en-Y cystojejunostomy | Perforated pancreatic pseudocyst with sock |
|  | Laparoscopy converted to laparotomy for adhesive small bowel obstruction | Adhesive small bowel obstruction |
|  | Hartmann’s procedure | Large bowel obstruction |
|  | Roux-en-Y jejunostomy | Bouveret's syndrome |
|  | Laparotomy and small bowel resection | Small bowel obstruction |
|  | Relook laparotomy and washout and closure | Hinchey IV Diverticulitis |
|  | Laparotomy and open common bile duct exploration | Cholangitis and Reynold’s Pentad |
|  | Planned relook small bowel resection and small bowel anastomosis | Mesenteric ischemia |
|  | Open abdomen closure | Ischemic right colon |
|  | Reduction of small bowel volvulus and repair umbilical hernia | Small bowel obstruction |
| Patients 20–24 died. | | |
